# Supplementary material for: Predation and fragmentation portrayed in the statistical structure of prey time series
Source: BMC Ecol. 2009 May 6;9:10. doi: 10.1186/1472-6785-9-10 (PMC2689204; doi:10.1186/1472-6785-9-10)
Supplement: Additional file 2 — Voles and related classes ODDox Documentation. ODDox documentation of the agent-based model (ALMaSS) applied by Hendrichsen et al. The documentation is started by activating main.html. [file 1472-6785-9-10-S2.zip › Vole_ODDox/_predators_8_h.html]

ALMaSS ODDox: Predators.H File Reference

- Main Page
- Related Pages
- Classes
- Files

# Predators.H File Reference

---

## Detailed Description

**The header file for all predator lifestages and population manager classes**

Version of 28 January 2001.   
By Chris J. Topping

`#include <vector>`  
`#include "ALMaSS_Setup.h"`  
`#include "../Landscape/ls.h"`  
`#include "../BatchALMaSS/PopulationManager.h"`  
`#include "GeneticMaterial.h"`  
`#include "Vole_all.h"`  

|  |  |
| --- | --- |
|  |  |
| Classes | |
| class | Owl |
|  | The Owl class is one of two current implementations of TPredator. More... |
| class | struct\_Predator |
|  | Used for creation of a new predator object. More... |
| class | TPredator |
|  | The base class for predators encompsassing all their general behaviours. More... |
| class | TPredator\_Population\_Manager |
|  | The class to handle all predator population related matters. More... |
| class | Weasel |
|  | The Weasel class is one of two current implementations of TPredator. More... |
| Typedefs | |
| typedef vector< TPredator \* > | TListOfPredators |
| Enumerations | |
| enum | TTypeOfPredatorState { tops\_InitialState = 0, tops\_Hunting, tops\_Dispersal, tops\_Movement } |

---

## Typedef Documentation

typedef vector<TPredator\*> TListOfPredators

Used for the population manager's list of predators

---

## Enumeration Type Documentation

enum TTypeOfPredatorState

Predators like other ALMaSS animals work using a state/transition concept. These are the predator behavioural states.

**Enumerator:**
:   |  |  |
    | --- | --- |
    | *tops\_InitialState* |  |
    | *tops\_Hunting* |  |
    | *tops\_Dispersal* |  |
    | *tops\_Movement* |  |

```
00043 {
00044       tops_InitialState=0,
00045       tops_Hunting,
00046       tops_Dispersal,
00047       tops_Movement
00048 } TTypeOfPredatorState;
```

---

Generated on Thu Jan 22 14:13:45 2009 for ALMaSS ODDox by 
 1.5.6
